# Molecular Cloning and Characterization of* Babesia orientalis* Rhoptry Neck 2* Bo*RON2 Protein

**DOI:** 10.1155/2017/7259630

**Published:** 2017-07-09

**Authors:** Ngabu Malobi, Lan He, Long Yu, Pei He, Junwei He, Yali Sun, Yuan Huang, Junlong Zhao

**Affiliations:** ^1^State Key Laboratory of Agricultural Microbiology, College of Veterinary Medicine, Huazhong Agricultural University, Wuhan, Hubei 430070, China; ^2^Key Laboratory of Animal Epidemical Disease and Infectious Zoonoses, Ministry of Agriculture, Huazhong Agricultural University, Wuhan, China

## Abstract

Babesiosis caused by* Babesia orientalis* is one of the most prevalent infections of water buffalo transmitted by* Rhipicephalus haemaphysaloides* causing a parasitic and hemolytic disease. The organelles proteins localized in apical membrane especially rhoptries neck and microneme protein form a complex called moving junction important during invasion process of parasites belonging to apicomplexan group, including* Babesia* species. A truncated fragment coding a 936 bps fragment was cloned in pMD-19T and subcloned into pET32 (a)^+^ expression vector, expressed in* E. coli* BL21. Purified recombinant* Bo*RON2 was used to produce polyclonal antibody against* Bo*RON2. Here, we identified the full sequence of gene encoding the rhoptry neck 2 protein that we named* Bo*RON2 which is 4035 bp in full-length open reading frame without introns, encoding a polypeptide of 1345 amino acids. Western blot of r*Bo*RON2 probed with buffalo positive serum analysis revealed a band of around 150 kDa in parasite lysates, suggesting an active involvement during invasion process. These findings most likely are constructive in perspective of ongoing research focused particularly on water buffalo babesiosis prevention and therapeutics and globally provide new information for genes comparative analysis.

## 1. Introduction

Protozoan parasites are a significant cause of mortality in human and animals worldwide. They belong to the phylum Apicomplexa, specifically characterized by the presence of complex specialized organelles at their apical end, and are responsible of important diseases, such as malaria which causes more than 1 million deaths worldwide, toxoplasmosis, cryptosporidiosis, coccidiosis, and babesiosis [1]. Babesiosis caused by* Babesia* genus causes a huge economic loss to the livestock industry worldwide [2, 3]. The life cycle of these organisms is very complex, involving different stages. In* Plasmodium* parasites causing malaria, for example, the first stage of infection starts by sporozoites injection into bloodstream by female* Anopheles* (infecting hepatics cells in mammals and salivary glands) followed by entry in liver cells and division named preerythrocytic stage, followed by the release of merozoites into bloodstream which initiate the asexual parasite multiplication stage, and these merozoites' differentiation leads to male and female gametocytes that invade mosquito midgut cells in which the gametocytes fusion produces a zygote that develops into a motile ookinete that penetrates the midgut wall and forms oocysts [4]. The oocyst enlarged over time and burst leading to sporozoites release which migrate to the mosquito salivary gland where they become susceptible of next infection through blood meal [5].

The parasite invasion in host cell is a crucial step in apicomplexan biology infection process, as their extracellular life is limited to a short period of time. This process is achieved in less than 10 seconds and is powered by the glideosome, a macromolecular complex consisting of adhesive proteins and an actomyosin system anchored in the inner membrane complex of the parasite [6]. This invasion involves sequential secretion of the contents of two secretory organelles from the apical complex: the micronemes and rhoptries [7]. Commonly the proteins localized in rhoptries and micronemes mediate interaction between host receptor and parasite during invasion [8]. From the apical complex, the parasite moves through this ring-like structure which is referred to as the moving junction (MJ) and whose function is to generate the parasitophorous membrane from the invaginated host plasma membrane [9]. The importance of rhoptries necks proteins in* P. falciparum* and in* T. gondii* as model for apicomplexan parasite [10] has raised concern in identifying the functional characterization of these proteins in other genera, such as* Neospora, Theileria, Babesia, Cryptosporidium, and other Plasmodium* species. The current model suggests that rhoptry neck protein 2 (RON2) attaches the moving junction to the host cell membrane via its predicted transmembrane domains serving as a receptor for apical membrane antigen 1 (AMA-1), which anchors RONs proteins of junction complex to the parasite surface [10], proving the high importance of rhoptry neck 2 protein function in apicomplexan parasites group.

Previous studies on* T. gondii* identified four rhoptries neck proteins (RON2, RON4, RON5, and RON8) found to be exported from apical rhoptries forming a complex at the host parasite interface [10–12]; however in* Plasmodium* only the 3 orthologues RON2, RON4, and RON5 found in* Toxoplasma* have been identified [13, 14]. The micronemal protein AMA-1 plays an important role during the tight junction formation with rhoptries proteins and anchors the merozoite to host surface cell leading to the invasion [15]; additionally, although the invasion inhibitory of AMA-1/RON2 interaction has been proved to be crucial for* T. gondii* and* P. falciparum* from preventing entry in their respective host cell, the intraspecies interaction is conserved [16].

Among* Babesia* species, recently, the proteins RON2 of* B. microti* and* B. divergens* were reported to be also homologues to other RON2 proteins of apicomplexan and the purified IgG from* B. divergens* RON2 antibodies was shown to be able to inhibit the invasion over 36 hours up to 44% [17]. However, in* B. orientalis*, AMA-1 protein was identified and has showed to have a similar and relative conservation of domain three (DIII) and the presence of four cysteine motifs among* Babesia* species [18]; thus the identification of* B. orientalis* RON2 protein and molecular cloning to evaluate the antigenicity of this protein are going to make a new step in order to understand this parasite infection and biochemical regulation mechanism.

## 2. Materials and Method

### 2.1. Merozoite Preparation

*B. orientalis* collected from water buffalo was cultured in our laboratory as described [19] and kept in nitrogen liquid [20]. The merozoite antigen of* B. orientalis* was prepared from blood infected by this parasite according to the modification saponin lysis method [21]. Erythrocytes from* B. orientalis* have been washed 3 times by using phosphate buffered saline (PBS) followed by suspension in 9 mL of red blood cells (RBC) lysis buffer (Tiangen, China) and incubation at 37°C for 5 min. After solubilization, the antigen was clarified by centrifugation at 10,000 ×g for 1 h, resuspended in 1 mL PBS, and then stored at −20°C for further use.

### 2.2. Genomic DNA and cDNA Preparation

For DNA and RNA extraction, we collected blood sample from experimentally infected water buffalo with 3% parasitemia into EDTA tubes (Qingdao Pharmacypro Co., Ltd.). Genomic DNA (gDNA) was isolated by using QIAmp DNA Mini Kit™ (Qiagen Hilden, Germany) following the manufacturer protocol. The collected gDNA was stored at −20°C for further use. Total RNA was extracted from 250 *μ*L of red blood cells by using TRIzol RNA extraction kit (Invitrogen, USA); RNase inhibitor (RNase OUT recombinant ribonuclease inhibitor Invitrogen, USA) was added; then the cDNA was synthesized by reverse transcription using FastQant RT Kit (Tiangen Biotech (Beijing) Co., Ltd.).

### 2.3. Identification, Amplification, and Sequencing of* Bo*RON2

The degenerate primer was designed through the consensus sequence found using ClustalW [22] from alignment based upon similarity of related species published RON2 of* B. bovis* (XM_001608765.1),* B. bigemina* (KU696964.1),* B. divergens* (GU 198499.2), and* B. microti* (XM_01279409.1) nucleotides sequences from NCBI. The amplified PCR product supposed to represent the partial* B. orientalis* RON2 was separated in 2% agarose gel ethidium bromide and cleaned using QIAquick gel extraction kit (Qiagen, China) and cloned into pMD-19T cloning vector (Takara Biotechnology, China) for sequencing. The partial amplified sequence was identified in a cDNA library contig in the complete genome sequence of* B. orientalis* (unpublished), and then the complete open reading frame was identified by overlapping contigs. By using nBLAST search, the sequence has been confirmed as RON2 regarding the high similarity with its orthologues. The complete sequence was obtained by another PCR amplification using a specific pair of primers (Table 2) and the resulting product was sequenced for confirmation.

### 2.4. Sequence Analysis

The obtained* Bo*RON2 sequence was analyzed by using bioinformatic tools. SP.4.1 software was used to predict the presence of signal peptide [23]. The transmembrane domain was predicted with TMHMM v.2.0 server [24] and low complexity regions were analyzed by using SMART [25]. Alpha helix and beta sheet were predicted by using Psi Pred server [26]. The presence of repeat was assessed by the sequence tandem repeat using the modeling software XSTREAM [27]. Mega 6 was implemented for phylogenetic tree construction [28]. The tertiary dimensional structure was predicted by using I-Tasser standalone package [29]. For identification of probable anchor location motif scan has been used through Expasy server (http://prosite.expasy.org/). ClustalW was used for alignment and manual correction was applied by using Bioedit [22, 30].

### 2.5. Recombinant Expression and Antibody Production

The gDNA fragment coding a C-terminal region which represents *Bo*RON2_914–1226_ peptide was amplified to produce a polyclonal antibody by using the expression vector p ET-32(a)+ to generate a recombinant His tag RON2 protein within* SalI* and* BamhI* restrictions sites enzymes underlined with a specific primer (Table 2). After successful sequencing the resulting plasmid was transformed into bacteria* E. coli* BL21 strain (Transgen, China). The recombinant protein was expressed after inducing 16 hours at 18°C with IPTG and visualized through SDS-PAGE. The protein expressed as inclusions bodies was solubilized in 8 M urea and eluted at 150 mM via His tag protein pure Ni-NTA resin charged column following the manufacturer's instruction (Transgen, China). BCA protein assay kit (Beyotime Institute of Biotechnology, China) was used to determine the protein concentration. To generate antirecombinant* Bo*RON2-C serum, 2 rabbits were immunized subcutaneously with 500 *μ*g of r*Bo*RON2-C emulsified in Freund's complete adjuvant (Sigma, USA) first time at day 0 and then emulsified in Freund's incomplete adjuvant (FIA) the next 4 times, respectively, at days 20, 35, 45, and 60 followed by antisera collection.

### 2.6. Western Blot Analysis

The reactivity of* Bo*RON2-C protein for immune response was analyzed by western blot with* B. orientalis* erythrocytes infected serum and His tag antibody used as positive control and normal serum as negative control. The recombinant* Bo*RON2-C was subjected under reducing condition to 12% SDS-PAGE analysis and transferred to a membrane nitrocellulose (Millipore USA) and then blocking buffer for fixation (1% BSA in TBS with 5% tween) followed by 2 h incubation moderately by shaking at room temperature and probed with secondary antibody Ig rabbit anti-goat. To identify the native* Bo*RON2-C in merozoite, rabbit antisera collected from r*Bo*RON2-C immunized rabbit was reacted with parasite lysates subjected to SDS-PAGE 12% followed by transfer electroblotting onto nitrocellulose membrane and then probed with rabbit polyclonal antiserum at 1 : 500 dilutions overnight at 4°C in TBST, after 5 times of washing with TBST, the secondary conjugate rabbit anti-goat antibody was added as final incubation.

## 3. Result

### 3.1. Molecular Characterization and Bioinformatic Analysis

**Figure 1:**
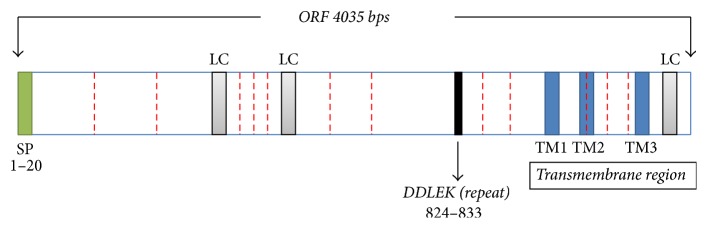
Schematic representation of* Babesia orientalis* RON2 gene with an open reading frame of 4035 bps length, signal peptide is shown is green (1–20), the three transmembrane (TM) domains are shown in blue, in grey are the predicted low complexity (LC) regions, and in red are the twelve cysteine residues contained in that gene. The repeat motif is shown in black between the amino acid regions 824 and 833.

**Figure 2:**
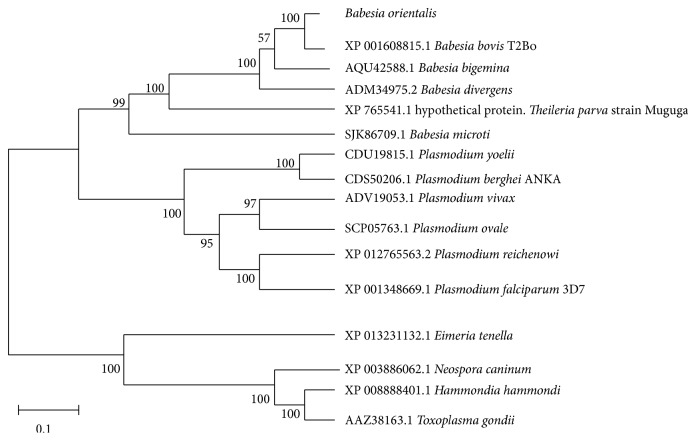
*Relationship of BoRON2 with other apicomplexan related RON2 proteins*. The neighbor joining tree was constructed using protein sequence of* Babesia orientalis* RON2 with its homologues found in NCBI, and the respective accessions numbers are indicated. The scale bar shows the length of branch; every 0.1 nucleotides' difference per 100 is represented.* B. orientalis* RON2 is in the same clade with* B. bovis*,* B. bigemina*, and* B. divergens*, more close to* B. bovis*.

**Figure 3:**
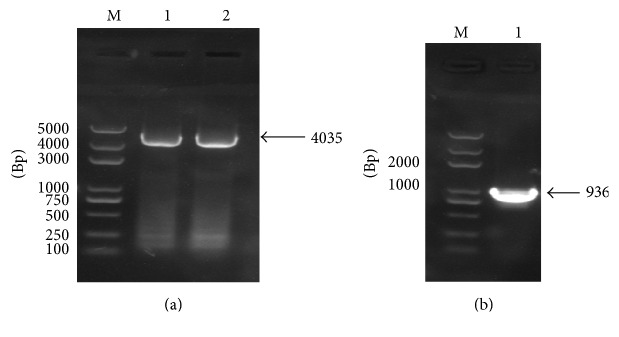
*PCR amplification of rhoptry neck 2 gene of Babesia orientalis from gDNA and cDNA and amplification of fragment used for polyclonal antibody production*. (a) M: nucleotide marker (15000 bps), line 1: amplification of* Bo*RON2 genomic DNA open reading frame showing a specific band of 4035 bp, and line 2: amplification of cDNA showing to have the same size with genomic DNA. (b) Line 1, the amplicon of product used for polyclonal antibody.

The obtained gDNA of* Babesia orientalis* was amplified and the complete RON2 gene sequence was identified by using specific primer as indicated on Table 2.* Bo*RON2 gene is 4035 bp nucleotides in length, encoding for 1345 amino acid residues with one copy of imperfect repeat sequence one time repeated in positions 824–833 (DDLEK–DDSEK). The amino acid sequence contains 12 cysteine residues as shown in Figure 1 and was predicted 150 kDa by using a computer based molecular weight calculator [31]. The phylogenetic tree shows that the more close species based on alignment of* Bo*RON2 protein as query is* B. bovis* (T2Bo strain) RON2 protein belonging into the same clade (Figure 2). They share 72% similarity, followed by* B. bigemina* with 70% of similarity. The amplification of cDNA has shown an amplicon of similar size with gDNA on gel electrophoresis (Figure 3(a)). Finally, the sequencing of cDNA has confirmed the intronless of this gene. The phylogenetic tree of* Bo*RON2 protein sequence with homologues was performed to identify the distance between them. The neighbor joining tree shows values of each branch with 0.1 as the length of branch (Figure 2). The protein has 7 conserved cysteine residues among aligned apicomplexan orthologues RON2, and 4 of them are located in C-terminal region alternating with transmembrane domains (Figure 5). In fact, many molecular relevant malaria antigens, actively functional, contain cysteine motifs in their structure forming disulfides bridges and/or occasioning three-dimensional structures fulfilling their biological function [32]. In* P. falciparum* the protein rhoptry neck 6* Pf*RON6 is conserved among apicomplexans except for rodent malaria orthologue, and rich cysteine region was shown to be involved in parasite survival function [33]. The molecular scanning has revealed a putative phosphotidylinositol specific lipase domain at amino acid positions 1026 to 1086 probably involved in membrane trafficking during parasite invasion and a putative anchoring site during infective process.* Bo*RON2 protein has a putative signal peptide within its 20 first amino acids characterized by the presence of three transmembrane domains all located toward the C-terminal region between residues 1110 and 1251 and three low complexity regions. The relative conservation in C-terminal fragment covering the region of amino acid comprising between 1018 and 1295 (Figure 5) is most likely due to the presence in that region of active site for* B. orientalis* RON2 interaction and important domains during the invasion process. Neighbor joining phylogenetic tree was built from the protein sequence of* B. orientalis* RON2 submitted on GenBank with closely related apicomplexan parasites species as indicated with respective accession number (Table 1) which was obtained by using protein search on NCBI. The tertiary structure sequence is predominantly dominated by the presence of alpha helix (Figure 6) without coiled coils motifs structure in contrary to some reported RON2 orthologues such as* P. falciparum* and* P. vivax* [34], which may explain one of the host specificity reasons.

### 3.2. *Bo*RON2 Immunoblotting Identification

**Figure 4:**
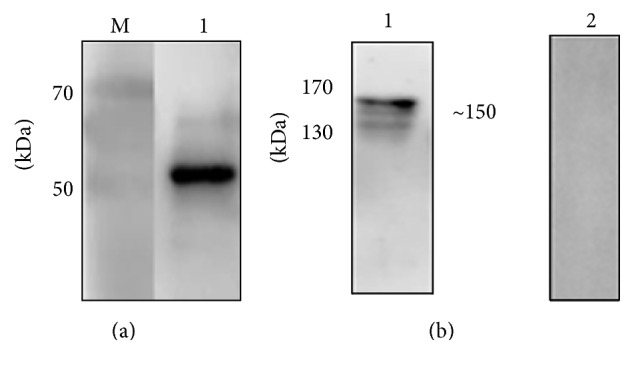
*Western blot of recombinant protein BoRON2-C*. (a) A specific band of 53 kDa was detected in reaction of recombinant* Bo*RON2 with anti* B. orientalis* water buffalo serum on line 1 and line M the prestained protein marker. (b) Immunoblot for detection of* Bo*RON2 protein in parasite lysates. Line 1, a unique band of around 150 kDa was detected in parasite lysate probed with polyclonal* Bo*RON2 antibody; line 2: no band detected with preimmune serum in parasite lysates.

**Figure 5:**
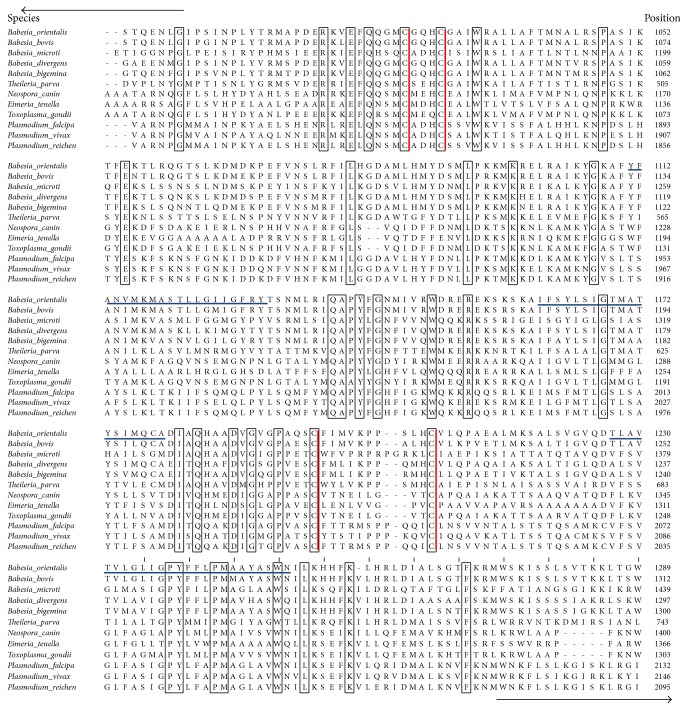
*Amino acid alignment of Babesia orientalis RON2 and other orthologues*. The alignment showing only C-terminal the most conserved region (flanking the region of AA sequence comprising between 1018 and 1295) was generated by using ClustalW. The conserved residues sequences are drawn in black boxes and the four conserved cysteine in C-terminal region in red line among the all proteins RON2 sequences aligned and the three predicted transmembrane regions of* Bo*RON2 are underlined in blue, the 2 arrows indicating the 5′ and 3′ direction of sequence.

**Figure 6:**
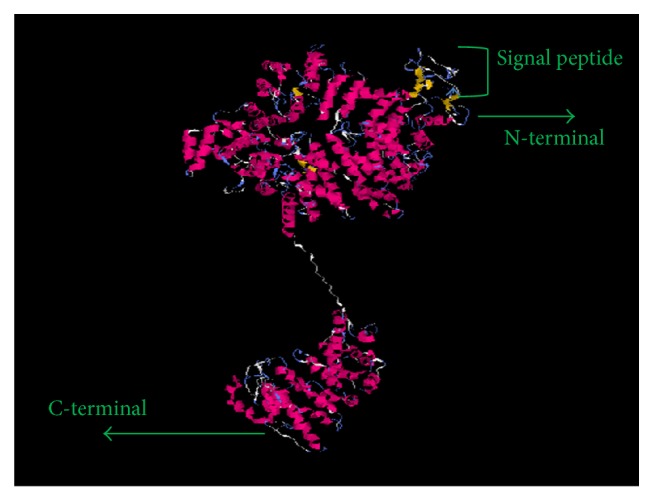
*Babesia orientalis RON2 tertiary prediction structure*. The predicted model with the highest C-score of* B. orientalis* RON2 protein.

The recombinant* Bo*RON2 antibody from amplification of 936 bp (Figure 3(b)) located between the amino acid regions 914 and 1226 was expressed as His Thioredoxin tag fusion native protein. A strong band of 53 kDa was detected when probing the recombinant* Bo*RON2 with positive* B. orientalis* serum of water buffalo (Figure 4(a)). The collected sera from rabbits immunized with* Bo*RON2-C antigen have been used for* B. orientalis* RON2 protein identification in parasite lysates through immunoblot assay. As indicated on Figure 4(b) a band at ~150 kDa in parasite lysates was observed consistent with the computational predicted size, but no band was observed with the preimmune serum (PI).

## 4. Discussion

Apicomplexan phylum contains obligate intracellular parasites characterized by a particular process during host cell invasion involving the secretory organelles essentially rhoptries and micronemes proteins [35]. They form a distinctive structure called moving junction, in the space between host plasma membrane and parasite [33]. Most of these proteins of moving junction identified in* T. gondii* have been found to be well conserved among apicomplexan parasites, with clear homologues identified in other species such as* Plasmodium* [36]. The rhoptries neck protein 2 has been shown to be conserved among different reported homologous species [37]. Numerous studies previously have reported both in* Plasmodium* and in* Toxoplasma* the direct interaction between AMA-1 and RON2 [38], and the occurrence of RON4 in homologues* Plasmodium* sp. and* Babesia* sp. has suggested the conservation of moving junction components across apicomplexan phylum [12].

In this work, we reported the RON2 protein of* B. orientalis*, which has been shown to be conserved to reported homologues, signifying the importance of this protein in biology of this parasite in infection stages. To our knowledge, this study is the first work to report molecular identification and characterization of* B. orientalis* RON2. The recombinant polyclonal antibody* Bo*RON2 raised was able to detect a unique band at ~150 kDa in parasite lysates, which was consistent with the predicted size. As reported previously, the generated polyclonal antibody RON2 of* B. microti* was able to detect a ~170 kDa predominant band consistent to the expected size and a product at ~55 kDa with no clear identity, but* B. divergens* antibodies identified also a consistent dominant band at ~170 kDa and 2 secondary bands at ~130 kDa and ~60 kDa [17]. Furthermore, in study on* P. falciparum* RON2 protein, sera in schizont stage have reacted with a band larger than 250 kDa corresponding to the predicted size and an additional band of 80 kDa without specific identity, but probably a Pf RON2 processing product [39]. However* P. vivax* RON2 polyclonal antibody has detected 2 bands (~220 kDa and ~185 kDa) different to the expected size (240 kDa) suggesting not only a proteolytic process but also a particular behavior during SDS-PAGE running [34]; curiously the same behavior for* T. gondii* RON2 characterized by an abnormal migration was observed, although the polyclonal antibody* T. gondii* RON2 has detected a band less than the predicted size (~150 kDa) cleaved to generate a 120 kDa protein [10].

The phylogenetic and BLAST analysis indicate the closeness of taxonomical relationship with other apicomplexan rhoptries proteins RON2, confirming, like for many reported* B. orientalis* genes such as AMA-1, Hsp20, Cox1, and Cob genes, that* B. bovis* is phylogenetically the closest among all related apicomplexan parasites with 72% sequence similarity; additionally the apicoplast genome is more similar to* Babesia bovis* apicoplast genome structure [40]. The analysis of cDNA and gDNA has revealed that there are no introns. It has been also reported for AMA-1 nucleotide sequence of* B. orientalis* the absence of introns [17], supposing likely that the splicing process involving the apical complex proteins in this genus is a probable inexistent event during protein translation for invasion establishment process. The signal peptide is followed by N-terminal region relatively not conserved harboring 2 low complexity domains needed to be analyzed for functional identification. We noticed the presence of 12 cysteine residues; three between the two hydrophobic regions close to N-terminal region are conserved. The conserved C-terminal region contains 4 conserved cysteine residues, supposing the metabolic importance of that region in this genus; in fact most of apicomplexan parasites RON2, the cysteine-rich globular domain in merozoite surface, or apical proteins are involved in crucial interaction between ligand and surfaces receptors and also during invasion between host and parasite [41–43]. Additionally in* P. falciparum* merozoite and* T. gondii* tachyzoite the C-terminal recombinant protein RON2 was able to block the host cell invasion [15]. The relative conservation of* Babesia orientalis* C-terminal region presumes most likely the active site for interaction with AMA-1 facilitating this parasite invasion.

## 5. Conclusion

The underlying mechanism of buffalo babesiosis caused by* B. orientalis *in molecular level passes inevitably through RON2 protein identification. Herein we reported* B. orientalis* RON2 which has been identified to be conserved among related species, with a probable C-terminal physiologically and relatively more active. The abundance of cysteine residue is most likely relevant to its function during invasion, which unlikely makes this protein hard for solubilization in active form during experiment for the expression in bacteria. The polyclonal serum has been able to detect the native protein in parasite lysates suggesting* Bo*RON2 as a good potential candidate for vaccine and diagnosis of disease. Therefore, the limitation of doing more experiments for biochemistry and physiology purposes is due to the complication of culturing in vitro* B. orientalis* parasite.

## Supplementary Material

Supplemental Figure 1: Nucleotide sequence alignment of B. orientalis close related species to find the consensus sequence for degenerate primer design. Supplemental Figure 2: Partial amplified sequence from degenerate primer designed.

## Figures and Tables

**Table 1:** Apicomplexan species used for alignment and phylogenetic tree.

Organism	Accession number
*Babesia bovis*	XM_001608815.1
*Babesia bigemina*	AQU42588.1
*Babesia microti*	SJK86709.1
*Babesia divergens*	ADM34975.2
*Theileria parva*	XP_765541.1
*Neospora caninum*	XP_003886062.1
*Toxoplasma gondii*	AAZ38163.1
*Eimeria tenella*	XP_013231132.1
*Plasmodium vivax*	ADV19053.1
*Plasmodium falciparum*	XP_001348669.1
*Plasmodium reichenowi*	XP_012765563.2
*Babesia orientalis*	To be submitted
*Hammondia hammondi*	XP_008888401.1
*Plasmodium ovale*	SCP05763.1
*Plasmodium berghei*	CDS50206.1
*Plasmodium yoelii*	CDU19815.1

**Table 2:** Set of primers used for amplification in this work.

Primer	Sequences (5′-3′)	Remarks
F0	TGGATGMAATGATTTCTGGACCC	Degenerate primer
R0	ACGCACGTTTCCACTTGTTGT	
F1	ATGTTTGCGGTTACCCTGGCAACGATCACACTAGTG	ORF
R1	TCAATTAAATACAGTGTATGAGAAGTTGTCGTCAGC	
Fa	CGGGATCCATGGCGTTCCAAAGAGCTGCTA	Antibody production
Ra	GCGTCGACTCATTGTACACCTACTGAGAGTGC	

## References

[1] Liu L., Johnson H., Cousens S. (2012). Global, regional and national causes of child mortality: an update systematic analysis for 2010 with time trends since 2000. *The Lancet*.

[2] Brown W. C., Palmer G. H. (1999). Designing blood-stage vaccines against Babesia bovis and B. bigemina. *Parasitology Today*.

[3] Dewaal D. T. (2000). Global importance of piroplasmosis. *The Journal of Protozoology*.

[4] Bousema T., Okell L., Felger I., Drakeley C. (2014). Asymptomatic malaria infections: Detectability, transmissibility and public health relevance. *Nature Reviews Microbiology*.

[5] Stone W. J. R., Eldering M., Van Gemert G.-J. (2013). The relevance and applicability of oocyst prevalence as a read-out for mosquito feeding assays. *Scientific Reports*.

[6] Opitz C., Soldati D. (2002). A dynamic complex powering gliding motion and host cell invasion by Toxoplasma gondii. *Molecular Microbiology*.

[7] Carruthers V. B., Sibley L. D. (1997). Sequential protein secretion front three distinct organelles of Toxoplasma gondii accompanies invasion of human fibroblasts. *European Journal of Cell Biology*.

[8] Gaur D., Chitnis C. E. (2011). Molecular interactions and signaling mechanisms during erythrocyte invasion by malaria parasites. *Current Opinion in Microbiology*.

[9] Suss-Toby E., Zimmerberg J., Ward G. E. (1996). Toxoplasma invasion: The parasitophorous vacuole is formed from host cell plasma membrane and pinches off via a fission pore. *Journal of Cell Biology*.

[10] Straub K. W., Cheng S. J., Sohn C. S., Bradley P. J. (2009). Novel components of the Apicomplexan moving junction reveal conserved and coccidia-restricted elements. *Cellular Microbiology*.

[11] Alexander D. L., Mital J., Ward G. E., Bradley P., Boothroyd J. C. (2005). Identification of the moving junction complex of Toxoplasma gondii: a collaboration between distinct secretory organelles.. *PLoS Pathogens*.

[12] Lebrun M., Michelin A., El Hajj H. (2005). The rhoptry neck protein RON4 relocalizes at the moving junction during Toxoplasma gondii invasion. *Cellular Microbiology*.

[13] Alexander D. L., Arastu-Kapur S., Dubremetz J.-F., Boothroyd J. C. (2006). Plasmodium falciparum AMA1 binds a rhoptry neck protein homologous to TgRON4, a component of the moving junction in Toxoplasma gondii. *Eukaryotic Cell*.

[14] Collins C. R., Withers-Martinez C., Hackett F., Blackman M. J. (2009). An inhibitory antibody blocks interactions between components of the malarial invasion machinery. *PLoS Pathogens*.

[15] Richard D., MacRaild C. A., Riglar D. T. (2010). Interaction between *Plasmodium falciparum* apical membrane antigen 1 and the rhoptry neck protein complex defines a key step in the erythrocyte invasion process of malaria parasites. *The Journal of Biological Chemistry*.

[16] Lamarque M., Besteiro S., Papoin J. (2011). The RON2-AMA1 interaction is a critical step in moving junction-dependent invasion by apicomplexan parasites. *PLoS Pathogens*.

[17] Ord R. L., Rodriguez M., Cursino-Santos J. R. (2016). Identification and characterization of the rhoptry neck protein 2 in Babesia divergens and B. microti. *Infection and Immunity*.

[18] He L., Fan L., Hu J. (2015). Characterisation of a Babesia orientalis apical membrane antigen, and comparison of its orthologues among selected apicomplexans. *Ticks and Tick-borne Diseases*.

[19] He L., Feng H.-H., Zhang Q.-L. (2011). Development and evaluation of real-time PCR assay for the detection of babesia orientalis in water buffalo (Bubalus bubalis, Linnaeus, 1758). *Journal of Parasitology*.

[20] Liu Z. L., Ma L. H., Yao B. A., Zhao J. L. (1995). Test of infected cattle by bite *Rhipicephalus haemaphysaloides* and injection with parasitized blood of buffalo with *Babesia*. *Acta Zootechnica Sin*.

[21] Conrad P. A., Iams K., Brown W. C., Sohanpal B., ole-MoiYoi O. K. (1987). DNA probes detect genomic diversity in Theileria parva stocks. *Molecular and Biochemical Parasitology*.

[22] Larkin M. A., Blackshields G., Brown N. P. (2007). Clustal W and clustal X version 2.0. *Bioinformatics*.

[23] Petersen T. N., Brunak S., Von Heijne G., Nielsen H. (2011). Discriminating signal peptides from transmembrane regions: signal P 4.0. *Nature Methods*.

[24] Krogh A., Larsson B., Von Heijne G., Sonnhammer E. L. L. (2001). Predicting transmembrane protein topology with a hidden Markov model: application to complete genomes. *Journal of Molecular Biology*.

[25] Letunic I., Doerks T., Bork P. (2012). SMART 7: recent updates to the protein domain annotation resource. *Nucleic Acids Research*.

[26] Buchan D. W. A., Minneci F., Nugent T. C. O., Bryson K., Jones D. T. (2013). Scalable web services for the PSIPRED protein analysis workbench. *Nucleic Acids Research*.

[27] Newman A. M., Cooper J. B. (2007). A practical algorithm for identification and architecture modeling of tandem repeats in protein sequences. *BMC Bioinformatics*.

[28] Tamura K., Stecher G., Peterson D., Filipski A., Kumar S. (2013). MEGA6: molecular evolutionary genetics analysis version 6.0. *Molecular Biology and Evolution*.

[29] Yang J., Yan R., Roy A., Xu D., Poisson J., Zhang Y. (2015). The I-TASSER suite: protein structure and function prediction. *Nature Methods*.

[30] Hall T. A. (1999). Bioedit: a user friendly biological sequences alignment and analysis program for windows 95/98/NT. *Nucleic Acids Symposium*.

[31] Gasteiger E., Hoogland C., Gattiker A., Wilkins M. R., Appel R. D., Bairoch A., Walker J. M. (2005). Protein identification and analysis tools on the expasy server. *The Proteomics Protocols Handbook*.

[32] Vulliez-Le Normand B., Tonkin M. L., Lamarque M. H. (2012). Structural and functional insights into the malaria parasite moving junction complex. *PLoS Pathogens*.

[33] Proellocks N. I., Kats L. M., Sheffield D. A. (2009). Characterisation of PfRON6, a plasmodium falciparum rhoptry neck protein with a novel cysteine-rich domain. *International Journal for Parasitology*.

[34] Arévalo-Pinzón G., Curtidor H., Patiño L. C., Patarroyo M. A. (2011). PvRON2, a new *Plasmodium vivax* rhoptry neck antigen. *Malaria Journal*.

[35] Aikawa M., Miller L. H., Johnson J., Rabbege J. (1978). Erythrocyte entry by malarial parasites. A moving junction between erythrocyte and parasite. *Journal of Cell Biology*.

[36] Dubremetz J. F., Garcia-Réguet N., Conseil V., Fourmaux M. N. (1998). Apical organelles and host-cell invasion by Apicomplexa. *International Journal for Parasitology*.

[37] Bradley P. J., Ward C., Cheng S. J. (2005). Proteomic analysis of rhoptry organelles reveals many novel constituents for host-parasite interactions in *Toxoplasma gondii*. *Journal of Biological Chemistry*.

[38] Shen B., Sibley L. D. (2012). The moving junction, a key portal to host cell invasion by apicomplexan parasites. *Current Opinion in Microbiology*.

[39] Cao J., Kaneko O., Thongkukiatkul A. (2009). Rhoptry neck protein RON2 forms a complex with microneme protein AMA1 in Plasmodium falciparum merozoites. *Parasitology International*.

[40] Huang Y., He L., Jifang H., He P., He J. (2015). Characterization and annotation of *Babesia orientalis* apicoplast genome. *Parasites & Vectors*.

[41] Goel V. K., Li X., Chen H., Liu S. C., Chishti A. H., Oh S. S. (2003). Band 3 is a host receptor binding merozoite surface protein 1 during the *Plasmodium falciparum* invasion of erythrocytes. *Proceedings of the National Academy of Sciences of the United States of America*.

[42] Li X., Chen H., Oo T. H. (2004). A co-ligand complex anchors plasmodium falciparum merozoites to the erythrocyte invasion receptor band 3. *Journal of Biological Chemistry*.

[43] Sim B. K. L., Chitnis C. E., Wasniowska K., Hadley T. J., Miller L. H. (1994). Receptor and ligand domains for invasion of erythrocytes by *Plasmodium falciparum*. *Science*.

