# Supplementary material for: Molecular Cloning and Characterization of Babesia orientalis Rhoptry Neck 2 BoRON2 Protein
Source: J Parasitol Res. 2017 Jul 9;2017:7259630. doi: 10.1155/2017/7259630 (PMC5523350; doi:10.1155/2017/7259630)

Additional file

#1

TGGATGCAAATGATTTCTGATCCTAGGAAGTATACATCTGTTTTCGAATACACCATTAAA
TACGACAACAGGATGTTTTCAGGAAAACGCAAATCCTGGATGGCTAATTACAAAAGTG
TTGAAAAGAAAGTCACACCTGGCTTAGATCTAATAAATCTCGATCTGCACACAGCTTTA
TCCATGCTCGATAACAAGGGCACCAACAAATACTCTTCGAAAAAAAGCCACCCTCTTA
AGAGGGCCTTAGTCTATATGTCTGCTTCGGGTATTAAACAATGGGTCGTTGGAAACCTC
GAAAACCTTCAAGAACGATTCGGATTTTCACCTTCAGGTCTCTTGGTTGGGAACCTAG
CACCATATTTCCGGCAAGTTGCGCAGAGCACCAGCGCTGGCCTTTGCCACATTTTCTTA
TACCACATGTTAACTTCGTTCAATCCTGCATATGAATTTGTAACGGACATTAGTGGTTTT
AGAGGGGGAAATCTGTTGTACAACATCGCTGAATCCTCGAACATGTTCATTCCAGCCA
GCCTAAAACGCGGAATCAAGTGGCTGCTAAAAGGTGGGTTAGCCAAGGAGTTTAGAC
GTGAAAAGGCCAAACACACCCTCCTACAATTGCTACCAGTCGAACTACTGAGGAAAG
CCATCAGCGCCATCACATTCGTAACTCATTCTCTCGCTGACATACAGATAAACCAAAAC
GCGGAAGTCTTTGGTCGTGGCTTACTTTCTGACAAGGACAGAATTAAAAAACATTTTAT
AAGTGGAGGATATGTTAACTATGTGGATAGTGTCATCAAGGAATGGTCTGATGAAGGAT
ATACGGAGGGCATAGCCAAGAAAGTTAAACAGGGCGATGATCTCAGTAAAGATGATTT
AGAGAAGGCTAATATGCACAAAATTGTACATACTGAATCGCTGAAATGGGAAAAGAAT
CTGAATTCGATAATATTGGAAGGGTACAATTCTTTCCTTGAGCTCCCTTCGATAAAGGTA
CTTGATGGGAAACACTCACTGATTTATGAGATTGTGAAGGATAGCAGGGACAATTTAGA
ACAGCATCTTAATGATACGATTTTTTTCGGTCGAGTTGTGAACCCACCTGCTTACAACA
ATAAGTGGAAACGGGCGT

In red: consensus sequences used for forward and reverse degenerate primers design.

1.forward

```
XM_012794094.1      TTGT-GGCAATTTGAACACTGGTAAAAAGGAGGCATTTCCG---GAATACTTGAGATTGA
XM_001608765.1      TACTCAGCGATGTAAAC-CTCGAC----AC-CGAGCAGAGAACCACGTGGATGCAAATGA
GU198499.2          TTCTGAGCGATGTTAGC-CTAGAT----GG-CGAAATTCCATACACATGGATGAAAATGA
KU696964.1          TCTTGAGTGATGTTAGC-CTGGAT----GG-GGAAATACCAACCACCTGGTTAAGGATGA
                    *  * .* .** * *.* ** *:     .   *.. : .   .. *. :*....:***

XM_012794094.1      TCTCTAACCCCTTCGAATACGGCGATATTTTTTCCATGACTATGTGGTG-----GGATCC
XM_001608765.1      TTTCTGACCCTAGGAAATACACGTCCAT-----CCTAGAGTATGCGATAAAATTTGACAA
GU198499.2          TTTCTGACCCCAGGAAATATGCCAGTAT-----ACTCGAATATACTCTTAAATATGACAA
KU696964.1          TTTCAGACCCTCGCAAGTATGCAACCGT-----GTTCGAATATGCAGTCAAATTCGACAA
                    * **:.****    .*.** .     .*       : ** ***.   *       ** ..
```

2.reverse

```
XM_012794094.1      TGTGTTCTTTGGCAAAGTGCTTCCCCC---AGGAAA-AACTAATAATGTTATTAAGAAAT
XM_001608765.1      TATTTTCTTCGGACGCGTTGTTAACCCACCCGAATACAACAACAAGTGGAAACGTGCGTT
GU198499.2          TGTTTTCTTCGGACGGGTTATACCGCCAACAGTCTATAACAACAAGTGGAAACGTGCGTT
KU696964.1          AGTGTTCTTTGGACGCGTGGTGCCACCGCCAGTTTATAACAACAAGTGGAAAAGGTTTTT
                    :.* ***** **... **  * .. **   .*  :* ***:*.:*.** :*: .    :*
```

1133 bases pairs length.

The nucleotide sequence flanking the region between 1611-2744.

The degenerate primer (Table 2)
We have used the approach consisting of identification a sequence in cDNA library from a contig of genome (unpublished *Babesia orientalis*) by designing a degenerate primer from the alignment of close related genes to find the consensus sequence that could amplify a fragment of BoRON2. If need I can send the alignment.

#2
The report on tandem repeat prediction

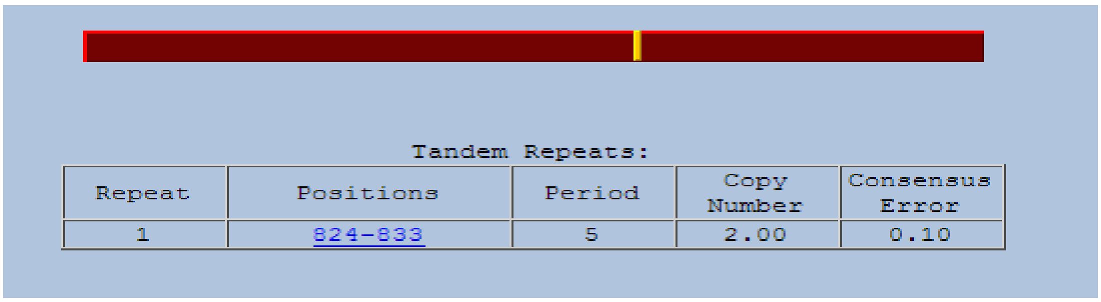

Tandem Repeats:

Repeat	Positions	Period	Copy Number	Consensus Error
1	824-833	5	2.00	0.10

```
DDLSK
DDLEK

DDLEK

 [D,2]LEK
```

3D predicted structure of *Babesia orientalis* RON2.

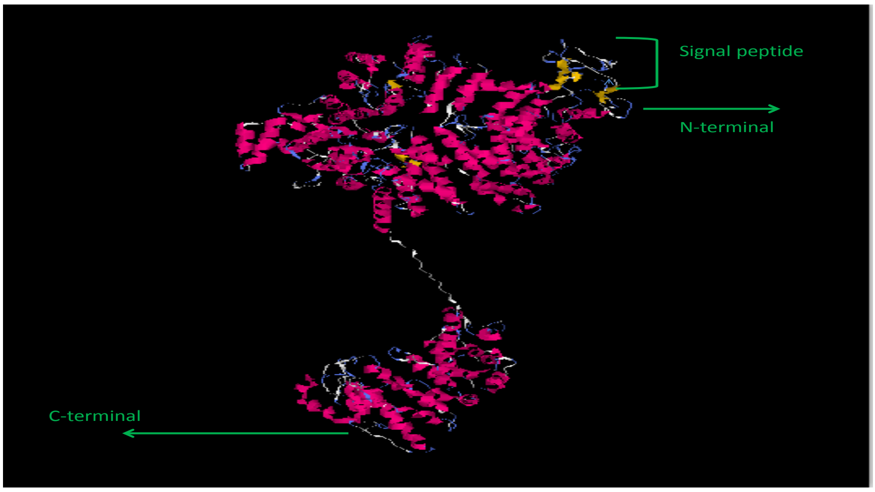

Supplement: Supplementary file 1 — Supplemental Figure 1: Nucleotide sequence alignment of B. orientalis close related species to find the consensus sequence for degenerate primer design. Supplemental Figure 2: Partial amplified sequence from degenerate primer designed. [file 7259630.f1.pdf]
